# *Mycoplasma pneumoniae* and *Chlamydia* spp. Infection in Community-Acquired Pneumonia, Germany, 2011–2012

**DOI:** 10.3201/eid2103.140927

**Published:** 2015-03

**Authors:** Roger Dumke, Christiane Schnee, Mathias W. Pletz, Jan Rupp, Enno Jacobs, Konrad Sachse, Gernot Rohde, CAPNETZ Study Group

**Affiliations:** Technical University Dresden Institute of Medical Microbiology and Hygiene, Dresden, Germany (R. Dumke, E. Jacobs);; Friedrich-Loeffler-Institut, Jena, Germany (C. Schnee, K. Sachse);; Jena University Hospital Center for Infectious Diseases and Infection Control, Jena (M.W. Pletz);; CAPNETZ STIFTUNG, Hannover, Germany (M.W. Pletz, J. Rupp, G. Rohde);; University of Lübeck, Lübeck, Germany (J. Rupp);; Maastricht University Medical Centre, Maastricht, the Netherlands (G. Rohde)

**Keywords:** community-acquired pneumonia, CAP, MLVA, bacterial pneumonia, outpatients, molecular diagnostics, Mycoplasma pneumoniae, Chlamydia pneumoniae, Chlamydia psittaci, Chlamydia, bacteria, Germany

## Abstract

*Mycoplasma pneumoniae* and *Chlamydia* spp., which are associated with community-acquired pneumonia (CAP), are difficult to propagate, and can cause clinically indistinguishable disease patterns. During 2011–2012, we used molecular methods to test adult patients in Germany with confirmed CAP for infection with these 2 pathogens. Overall, 12.3% (96/783) of samples were positive for *M. pneumoniae* and 3.9% (31/794) were positive for *Chlamydia* spp.; *C. psittaci* (2.1%) was detected more frequently than *C. pneumoniae* (1.4%). *M. pneumoniae* P1 type 1 predominated, and levels of macrolide resistance were low (3.1%). Quarterly rates of *M. pneumoniae*–positive samples ranged from 1.5% to 27.3%, showing a strong epidemic peak for these infections, but of *Chlamydia* spp. detection was consistent throughout the year. *M. pneumoniae*–positive patients were younger and more frequently female, had fewer co-occurring conditions, and experienced milder disease than did patients who tested negative. Clinicians should be aware of the epidemiology of these pathogens in CAP.

## Abstract 2

*M. pneumoniae* infections showed a strong epidemic peak, but *Chlamydia* spp. were consistently detected throughout the year.

Community-acquired pneumonia (CAP) is associated with high rates of illness and hospitalization; annual CAP incidence among adults in Europe has ranged from 1.5 to 1.7 per 1,000 population (*1*). Studies have shown that that a broad range of pathogens can cause CAP (*2*). Among these is *Mycoplasma pneumoniae*, a common agent of respiratory tract infections that is transmitted from person to person through aerosolization. The infection occurs in all age groups, but older children and young adults are affected at a higher frequency than other age groups. Clinical manifestations range from mild cases of tracheobronchitis to severe atypical pneumonia and can be followed by a broad spectrum of extrapulmonary complications. 

The epidemiology of *M. pneumoniae* infection is characterized by incidence peaks every 4–7 years; during these periods, *M. pneumoniae* is responsible for up to 25% of all cases of CAP (*3*). Between epidemic periods, proportions of 1%–8% are more typical (*4*). Reports from Europe and Asia have shown a notable increase in the frequency of infections caused by *M. pneumoniae* during 2011–2012 (*5*–*12*). 

For clarification of the epidemiology of *M. pneumoniae* infection and identification of the relevant periods of incidence peaks, molecular typing of the prevalent strains can be an efficient tool. *M. pneumoniae* strains can be divided into subtypes and variants according to sequence differences in the gene coding for the immunodominant main adhesin P1. It has been hypothesized that the specific antibody level in the host population can influence further infections and lead to a change of the dominating P1 type (*13*). The recently developed multilocus variable-number tandem-repeat (VNTR) analysis (MLVA) enables differentiation of strains with higher discriminatory power (*14*). Further studies are necessary to determine associations between P1 and MLVA typing. However, knowledge of the strain’s genotype identity currently has no therapeutic consequences. Because mycoplasmas, which do not have cell walls, are not susceptible to β-lactam antimicrobial drugs, macrolides are generally accepted as first-choice agents for treatment, especially in children. However, mutations in the 23S rRNA locus of *M. pneumoniae* have been shown to result in complete macrolide resistance (*15*). Resistance rates range from >90% in China (*16*) to <10% in Europe (*15*), requiring periodic monitoring of strains to identify possible new resistance or resistant strains.

*Chlamydia pneumoniae* is another agent associated with CAP that can also be involved in pharyngitis, bronchitis, and sinusitis. Reports have attributed 6%–20% of CAP cases to this bacterium (*17*), and its role in chronic respiratory illness (*18*) and exacerbation of asthma (*19*) has also been studied. *C. pneumoniae* infection is regarded as widely distributed, if not ubiquitous, with antibody prevalence rates >50% (*19*). The clinical course of infection varies from subclinical to mild and, more rarely, to severe manifestations of pneumonia. The outcome of infection is often dependent on the patient’s immune competence, but co-infection by other bacteria has been suggested to be relevant in 30% of adult cases of CAP (*20*). However, a discrepancy exists between the elevated serologic prevalence and the low figures obtained through DNA-based detection methods (*21*); a recent publication from Germany reported a prevalence <1% (*22*).

Other *Chlamydia* spp. have not usually been included in epidemiologic studies of pneumonia. In particular, *C. psittaci*, the causative agent of human psittacosis (or ornithosis), has not been investigated except in severe clinical pneumonia cases. As is the case for infections caused by by other chlamydiae, an asymptomatic or mild clinical course of *C. psittaci* is far more frequent than a fulminant outbreak of disease. Nevertheless, infections that do not result in overt illness may have long-term implications for the patient’s health, as was shown in cattle that were carriers of *Chlamydia* spp. but did not show signs of disease (*23*).

In this study, we used molecular diagnostic approaches to investigate the occurrence of *M. pneumoniae* and *Chlamydia* spp. in adult patients in Germany who had confirmed CAP. The use of molecular typing methods for *M. pneumoniae* in combination with the determination of macrolide resistance was intended to obtain a nationwide overview of circulating strains in a period of high incidence of infections. Parallel testing for *Chlamydia* spp. was included to explore the frequency of co-infections with 2 microorganisms that are difficult to propagate and that can cause disease patterns that may be clinically indistinguishable.

## Methods

### Patient Population, Samples, and Data Collection

The CAPNETZ study is a multicenter, prospective, epidemiologic cohort study initiated by the German Competence Network for Community-Acquired Pneumonia (http://www.capnetz.de [*24*]). The network comprises clinical centers throughout Germany representing hospitals and outpatient departments at all levels of health care provision that are involved in the management of CAP. The decision on timing and type of treatment for each patient is left to the discretion of the attending physician. No attempt is made to implement standardized criteria or rules for the assessment of pneumonia severity or for the decision to hospitalize.

For this study, we prospectively recorded all consecutive and nonselected patients who sought treatment for signs and symptoms of CAP during March 2011–December 2012. Eligible participants were adult patients (>18 years of age) who had CAP confirmed by a new pulmonary infiltrate on chest radiograph and >1 sign or symptom of lower respiratory tract infection (i.e., fever, cough, purulent sputum, focal chest signs). Exclusion criteria were the following: hospital admission within 28 days before sampling, presence of immunosuppression (defined as chemotherapy and/or neutropenia <1,000 10^6^/L during the previous 28 days), therapy with corticosteroids >20 mg for >14 days, known HIV infection, immunosuppressive therapy after organ or bone marrow transplant, or active tuberculosis. All patients gave written informed consent and received a pseudonym from an independent third party to ensure data security. The study is registered at the German Clinical Trial Register (DRKS-ID: DRKS00005274). 

All patients provided pharyngeal swab specimens for the determination of the presence of *M. pneumoniae* and *Chlamydia* spp. Follow-up consultations by phone call to patient or next of kin or family physician were conducted 28 days and 180 days after enrollment. All demographic, clinical, and diagnostic data for patients were recorded using standardized Web-based data sheets created by 2mt (Ulm, Germany). The study was approved by the Institutional Review Board of the Otto-von-Guericke University (Magdeburg, Germany) under ID 104/01 in 2001 and subsequently by all local institutional review boards.

### Sample Processing and Microbiological Investigations

#### DNA Extraction

Swab specimens were shipped overnight in transport medium to the Friedrich-Loeffler-Institut (Jena, Germany) for testing. DNA extraction was performed by using the High Pure PCR Template Preparation Kit (Roche Diagnostics, Mannheim, Germany) according to the manufacturer’s instructions.

#### Testing for *Chlamydia* spp.

To test for *Chlamydia* spp., we first conducted a real-time PCR specific for the family *Chlamydiaceae* (*25*). Positive samples were further examined by using a *C. psittaci*–specific real-time PCR (*26*) and a DNA microarray assay in ArrayStrip format that covered all *Chlamydia* spp., *Waddlia chondrophila*, and *Simkania negevensis* (*27*,*28*).

#### Testing for *M. pneumoniae*

Aliquots of the DNA extracts were examined by using a previously described real-time PCR assay targeting copies of the repetitive element RepMP1 (*29*). Positive samples were further tested for macrolide resistance by methods previously reported (*15*). P1 and MLVA type were determined by nested PCR approaches and sequencing (*30*,*31*).

### Statistical Analysis

Categorical data are presented as frequencies and were compared by χ^2^ or Fisher exact test, as appropriate. The Yates correction procedure was applied to all comparisons.

Continuous variables are presented as median and range. Differences were analyzed by using the Mann-Whitney U test; p values <0.05 were considered significant. All analyses were carried out in SPSS version 20 software (IBM/SPSS, Chicago, IL, USA).

## Results

Overall, 783 CAP patients were enrolled during March 2011–December 2012 and were tested for *M. pneumoniae*; 96 (12.3%) were positive. Patients who had *M. pneumoniae* infection were significantly younger and more frequently female, had fewer co-occurring conditions, and experienced significantly milder disease that did those who did not have *M. pneumoniae* infection (Table 1). *M. pneumoniae*–positive patients were more frequently pretreated with antimicrobial drugs; however, we found no significant differences in the classes of antimicrobial drugs administered, particularly not in the use of macrolides.

**Table 1:** Demographic and clinical characteristics of patients with community-acquired pneumonia whose respiratory tract samples were tested for *Mycoplasma pneumoniae*, Germany, 2011–2012*

Characteristic	All, n = 783	*M. pneumoniae*–positive, n = 96	*M. pneumoniae*–negative, n = 687	p value†
Median age, y (range)	61 (18–102)	39.5 (18–84)	64 (18–102)	<0.001
Male sex	56.8	43.8	58.7	0.008
BMI (range)	25.6 (13.9–56.5)	24.2 (18.8–44.5)	25.8 (13.9–56.5)	
Co-occurring conditions				
Chronic pulmonary disease	19.2	6.2	23.1	<0.001
Chronic renal disease	19.2	0	20.6	0.004
Chronic heart failure	26.1	11.3	27.2	
Diabetes mellitus	24.9	11.1	25.9	
Cerebro-vascular disease	9.5	3.7	9.3	
CURB scores‡				<0.001
0	57.3	77.0	54.7	
1	31.0	21.8	32.2	
2	10.5	1.1	11.8	
3	1.2	0	1.4	
4	0	0	0	
Antimicrobial drug pretreatment	29.3	51.0	26.2	<0.001
Macrolides	8.0	7.3	8.3	§
Mortality rate				
28 d	1.8	0	2.0	
180 d	4.6	0	5.2	

*Values are percentages except as indicated. BMI, body mass index. †Only significant values are shown. Comparison between *M. pneumoniae*-positive and -negative patients by χ^2^ or Mann-Whitney U test as appropriate. ‡Clinical evaluation of the following risk factors (each scores 1 point): Confusion; blood Urea >7 mmol/L; Respiratory rate ≥30 bpm; Blood pressure <90 (systolic) or ≤60 mm Hg (diastolic). §Also no significant differences for any other antimicrobial drug classes.

**Figure 1:**
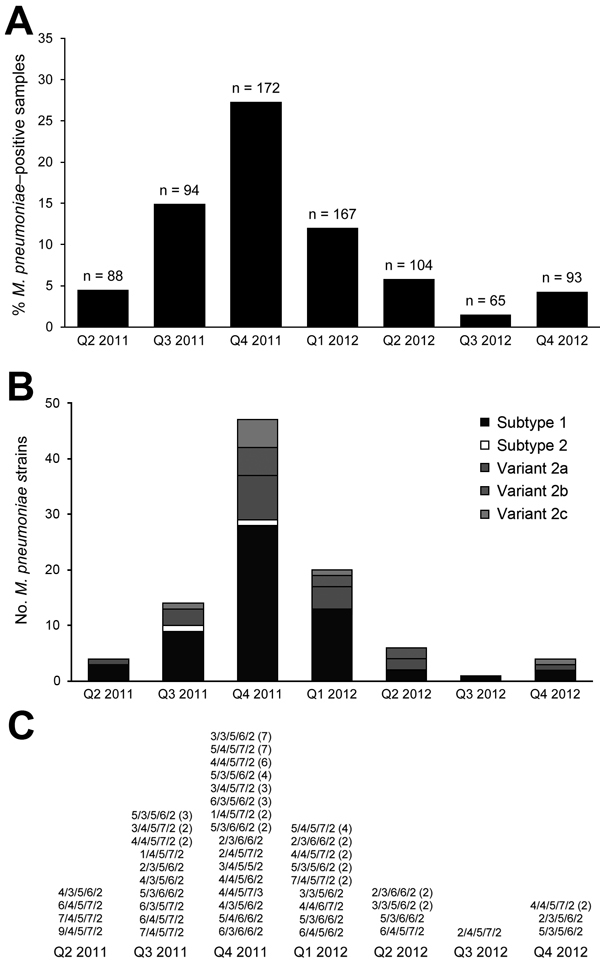
Results of molecular detection of *Mycoplasma pneumoniae* from 783 respiratory tract specimens from adult patients with pneumonia, Germany, March 2011–December 2012. A) Quarterly incidence of *M. pneumoniae* infection. n values indicate number of samples investigated by real-time PCR. B) *M. pneumoniae* P1 genotypes. C) *M. pneumoniae* multilocus variable-number tandem-repeat analysis types. Numbers of strains belonging to a given type are indicated in parentheses.

Using real-time PCR targeting the RepMP1 copies in the *M. pneumoniae* genome, we calculated a median of 7.8 × 10^3^ copies (range 4.1 × 10^1^ to 1.5 × 10^6^) in the *M. pneumoniae*–positive samples. *M. pneumoniae* positivity showed a clear season-dependent trend; low positivity of ≈4% was found at the beginning and the end of the investigation period, but high positivity was found during October 2011–December 2011. Quarterly incidence ranged from 1.5% (quarter 3, 2012) to 27.3% (quarter 4, 2011) (Figure 1). 

**Figure 2:**
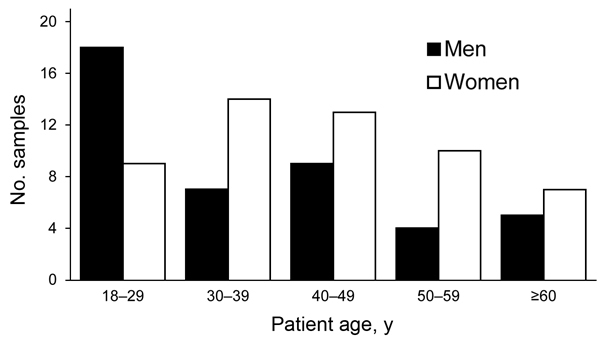
Age and sex distribution of patients with *Mycoplasma pneumoniae*–positive respiratory tract samples (n = 96), Germany, March 2011–December 2012. Percentage of positive samples for each age group: 18–29 y, 28.1%; 30–39 y, 21.9%; 40–49 y, 22.9%; 50–59 y, 14.6%; >60 y, 13.5%. (Total >100% due to rounding.)

The percentage of *M. pneumoniae*–positive patients from each age group ranged from 28.1% for the 18- to 29-year age group to 13.5% for the >60-year age group (Figure 2). The prevalence of *M. pneumoniae* decreased by age group: 18–29 years, 38%; 30–39 years, 31%; 40–49 years, 17%; 50–59 years, 13%; >60 years, 3%. More than half (55%) of *M. pneumoniae*–positive patients were female; only the 18- to 29-year age group had more *M. pneumoniae*–positive men than women.

Regarding the P1 genotype, all strains in the 96 *M. pneumoniae*–positive samples could be typed culture independently. Subtype 1 strains dominated (60.4%; Figure 1, panel B), followed by variant 2a (19.8%), variant 2b (9.4%), variant 2c (8.3), and subtype 2 strains (2.1%). A high proportion of subtype 1 strains were found during the entire 22-month investigation period, and all P1 types detected during the period were found at nearly the same proportion during the high-incidence period of October–December 2011.

Highly discriminatory MLVA was carried out on all positive samples using nested PCR. For 87 of the 96 samples, the complete recommended panel of 5 tandem-repeat regions could be amplified and sequenced successfully. Overall, 23 MLVA types were identified. The most common MLVA types were 4/4/5/7/2 (n = 12, 13.8%) and 5/4/5/7/2 (n = 11, 12.6%). During the high-incidence period (quarter 4 of 2011), the 42 *M. pneumoniae* strains that showed valid MLVA results belonged to 16 MLVA types. No clear correlation was found between P1 and MLVA typing. The 51 classifiable subtype 1 strains can be assigned to 16 MLVA types (Table 2). Five of these MLVA types can also be found in subtype 2 and the related variant 2 strains.

**Table 2:** Comparison of the results of P1 and MLVA typing of 87 *Mycoplasma pneumoniae* strains from patients with community-acquired pneumonia, Germany, 2011–2012

P1 type	MLVA types (no. strains)
Subtype 1, n = 51	1/4/5/7/2 (3), 2/3/6/6/2, 2/4/5/7/2 (2), 3/3/5/6/2 (2), 3/4/5/5/2 (2), 3/4/5/7/2 (5), 4/4/5/6/2 (2), 4/4/5/7/2 (11), 4/4/6/7/2, 5/3/5/6/2, 5/3/6/6/2, 5/4/5/7/2 (11), 6/3/5/7/2, 6/4/5/7/2 (3), 7/4/5/7/2 (3), 9/4/5/7/2
Subtype 2, n = 2	5/3/6/6/2 (2)
Variant 2a, n = 19	2/3/5/6/2 (2), 3/3/5/6/2 (6), 4/3/5/6/2 (2), 5/3/5/6/2 (5), 6/3/5/6/2 (2), 6/4/5/6/2, 7/4/5/7/2
Variant 2b, n = 8	2/3/6/6/2 (4), 5/3/6/6/2 (2), 5/4/6/6/2, 6/3/5/6/2
Variant 2c, n = 7	3/3/5/6/2 (2), 4/3/5/6/2, 5/3/5/6/2 (3), 6/3/5/6/2

*Underlining indicates MLVA types that occur in both subtype 1 and subtype 2/variant 2 strains. MLVA, multilocus variable-number tandem-repeat analysis.

On the basis of sequencing data, macrolide resistance can be assumed in 3 of the 96 *M. pneumoniae*–positive specimens (3.1%). All strains showed an A→G mutation at position 2063 of the 23S rRNA. The macrolide-resistant strains belonged to the predominant subtype 1 in P1 typing but differed in MLVA type (2/3/6/6/2, 2/4/5/7/2, and 5/4/5/7/2). The specimens containing resistant strains were sampled at different points of the study: December 2011, April 2012, and August 2012. All affected patients were female (ages 31, 42, and 42 years). For 2 of these patients, treatment with macrolides during the month before sampling was reported.

We further tested 794 patients for *Chlamydia* spp. (Table 3); 31 (3.9%) patients tested positive, 6 with dual infections (Table 4). In contrast to the *M. pneumoniae* findings, we found no significant differences in clinical characteristics between *Chlamydia*-positive and -negative patients (Table 3). Notably, no *Chlamydia*-positive patients received macrolides, whereas 8.3% of *Chlamydia*-negative patients did.

**Table 3:** Demographic and clinical characteristics of patients with community-acquired pneumonia whose respiratory tract samples were tested for *Chlamydia* spp., Germany, 2011–2012*

Characteristic	All, n = 794	*Chlamydia*-positive, n = 31	*Chlamydia*-negative, n = 763	p value†
Median age, y (range)	61 (18–102)	64 (18–89)	61 (18–102)	
Male sex	56.8	54.8	56.9	
BMI (range)	25.6 (13.9–56.5)	24.3 (17.7–45.3)	25.7 (13.9–56.5)	
Co-occurring				
Chronic pulmonary disease	21.2	32.3	20.7	
Chronic renal disease	19.2	0	19.9	p = 0.05
Chronic heart failure	25.7	12.5	26.2	
Diabetes mellitus	24.8	25.0	24.8	
Cerebro-vascular disease	9.3	12.5	9.2	
CURB scores‡				
0	56.8	54.8	56.9	
1	31.5	41.9	31.0	
2	10.5	3.2	10.8	
3	1.2	0	1.2	
4	0	0	0	
Antimicrobial drug pretreatment	29.1	25.8	29.3	
Macrolides	7.9	0	8.3	§
Mortality rate				
28 d	1.8	6.5	1.6	
180 d	4.7	6.5	4.6	

*Values are percentages except as indicated. BMI, body mass index. †Only significant values are shown. Comparison between *Chlamydia*-positive and -negative patients by χ^2^ or Mann-Whitney U test as appropriate. ‡Clinical evaluation of the following risk factors (each scores 1 point): Confusion; blood Urea >7 mmol/L; Respiratory rate ≥30 bpm; Blood pressure <90 (systolic) or ≤60 mm Hg (diastolic). §Also no significant differences for other antibiotic classes.

**Table 4:** Results of testing for *Chlamydia* spp. in 780 respiratory samples from patients with community-acquired pneumonia, Germany, 2011–2012

Species	No. (%) positive
*C. psittaci*	17 (2.2)
*C. pneumoniae*	11 (1.4)
*C. trachomatis*	3 (0.4)
*C. suis*	2 (0.3)
*C. abortus*	1 (0.1)
Other *Chlamydia* sp.	1 (0.1)
*Simkania negevensis*	1 (0.1)
*Waddlia chondrophila*	1 (0.1)
Total	37* (4.7)

*A total of 31 patients tested positive; 6 were infected with >1 *Chlamydia* species.

Test results identified *C. psittaci* (2.1%) as the most prevalent chlamydial species, followed by *C. pneumoniae* (1.4%). In addition, *C. trachomatis*, *Simkania negevensis*, and the animal pathogens *C. suis*, *C. abortus* and *Waddlia chondrophila* were identified in individual samples. The use of a DNA microarray assay combined with real-time PCR assays enabled us to detect multiple chlamydial infections; all 6 dual chlamydial infections (representing 19.3% of *Chlamydia*-positive patients) involved *C. psittaci*, 3 in conjunction with *C. pneumoniae* and 1 each with *C. abortus*, *S. negevensis*, and *W. chondrophila*. Co-infections with *M. pneumoniae* and *Chlamydia* spp. were detected in 3 samples (3.1% of *M. pneumonia*–positive and 8.1% of *Chlamydia*-positive specimens): 1 *M. pneumoniae* + *C. psittaci*, 1 *M. pneumoniae + C. pneumoniae*, and 1 *M. pneumoniae + C. psittaci + C. pneumoniae.*

## Discussion

Many studies have described the strictly time-dependent epidemiology of infections caused by *M. pneumoniae* (*3*). Although we did not include serologic testing to provide further information for the differentiation of colonization of patient from infection, the results of our study confirm a strong epidemic peak of *M. pneumoniae*–positive respiratory samples among adult patients with suspected CAP in Germany during 2011–2012. The incidence of infections temporarily rose to ≈27% during the fourth quarter of 2011, a level that is in accordance with other reports (*5*–*12*). Despite limited comparability (e.g., target used), the number of RepMP1 copies measured with real-time PCR (median 7.8 × 10^3^) is in the range of results of other studies (*32*,*33*). Because of the short duration of the epidemic peak and known deficiencies in testing routines for symptomatic *M. pneumoniae* patients, an increase in incidence could easily escape the notice of public health authorities. Moreover, because patients with *M. pneumoniae* infection were significantly younger than those without infection, it is possible that the true incidence might even be higher, given the fact that younger persons visit physicians less frequently and are admitted to hospitals less often than older patients. In addition, β-lactams are often used as the first-line antimicrobial drugs for CAP but are known to be inefficient in treatment of *Mycoplasma* infections; this conflict might represent another reason for the spread of this pathogen. In light of these results, revision of recent guidelines for management of CAP with antimicrobial drugs should be considered.

Typing of strains can help clarify the dynamics of epidemic peaks. There is no evidence that the incidence peak we registered was related to the genotype of circulating strains. *M. pneumoniae* is a genetically conserved organism, which implies limits to potential typing targets; most frequently used is P1, the main adhesin and most immunogenic protein, where sequence variation occurs mainly in the 2 copies of repetitive elements RepMP2/3 and RepMP4 of the P1-encoding locus *mpn141*. The epidemiologic importance of P1 genotypes is based on their ability to generate a specific host immune response (*13*). Therefore, P1 genotyping of circulating mycoplasma strains is helpful for understanding host-pathogen interactions and the infections ensuing. The current dominance of subtype 1 strains in combination with a rare occurrence of subtype 2 was also described in Europe and Asia (*11*,*34*). Subtype 2 strains have been replaced with the phylogenetically related variant 2 strains, which occurred in our study in different types. The variant 2c of this group was described in 2011 in several isolates from China (*34*); our detection of variant 2c strains in Germany confirms the parallel circulation of most variant 2 strains described this far. 

In this study, only particular regions of the P1 gene were selected for analysis, and the occurrence of additional P1 types showing further sequence variations cannot be ruled out. The recently characterized variant 2d (*35*) shares an identical 3′ part of the RepMP2/3 with variant 2a but differs in the 5′ region of the repetitive element; on the basis of those results, we retested all variant 2a strains, but we could not confirm the occurrence of variant 2d. 

For epidemiologic reasons, it is important that the period with a high proportion of *M. pneumoniae*–positive samples (October–December 2011) was not associated with a change of the dominating genotype nor with the presence of a particular P1 type. Previous reports have hypothesized that the circulation of genotype-specific antibodies in the human population can influence the number of infections and the dominating P1 type (*13*), but our data do not support this hypothesis.

In recent years, MLVA was introduced for typing of *M. pneumoniae* isolates (*14*) and extended to culture-independent typing from clinical samples (*31*). The determination of the number of 5 VNTR markers (Mpn1, 13–16) enables characterization of strains with a higher discriminatory power in comparison with P1 typing. Whereas the genomic regions used for MLVA are located mainly between genes and within genes of unknown function, the P1 protein plays a critical role in host–pathogen interaction. The most common MLVA types (4/4/5/7/2 and 5/4/5/7/2) we detected were also found in high abundance in strains recently identified in France, China, and the United States (*11*,*16*,*36*), thus indicating a worldwide dissemination of particular MLVA types. 

Regarding the strains included in our study, the assignment of P1 types to MLVA types confirmed that no clear relationship between the typing methods exists (Table 2). In agreement with the results of other reports (*11*,*14*,*16*), a low number of P1 subtype 1 strains belong to MLVA types that are typical for subtype 2 or variant strains (e.g., 5/3/5/6/2) and vice versa. In our opinion, both typing approaches are of practical importance and complement each other; the circulating P1 types reflect a more host-dependent pattern, whereas MLVA can differentiate strains with higher discriminatory power, enabling a better understanding of epidemiologic relationships. 

Instability of VNTR marker 1 has been reported (*36*), and the removal of Mpn1 from the current MLVA scheme has been suggested (*16*). For our data, the removal of Mpn1 would reduce the number of MLVA genotypes to 9, with >80% of strains belonging to 2 types, 3/5/6/2 and 4/5/7/2. This change would result in a substantial decrease of the discriminatory power of the MLVA typing method and would require efforts to include further VNTR markers showing a stable number of repeats within a given strain. 

The data from our study confirm that a nationwide peak of infections caused by *M. pneumoniae* is polyclonal, which is in agreement with results of other reports (*11*). In contrast, the clonal spread of *M. pneumoniae* can only be expected for small-scale endemic outbreaks with person-to-person transmission in close communities, such as families (*37*).

Since 2000, an increase of the worldwide occurrence of macrolide resistance in *M. pneumoniae* strains to 90% and higher has reported. Compared with data from Asia (*16*), the proportion of 3.1% of macrolide-resistant *M. pneumoniae* strains we detected is low. Results of studies from Germany in recent years have showed results in the same range (*15*), thus indicating a nearly constant prevalence of resistance. Nevertheless, resistant strains are circulating in the population we investigated, which requires further monitoring of strains to provide an updated overview of drug resistance. Several reports have confirmed that resistant strains were selected during antimicrobial drug treatment (*38*). However, in our study, increased prescription of macrolide antimicrobial drugs, which can be expected during a period of high CAP incidence, did not result in a measurable increase of resistant strains. In addition, because most current resistance data originate from pediatric patients, the results of this study are of general interest because we report on adult patients. 

Although further sequence differences of the 23S rRNA locus of *M. pneumoniae* have been described, the A→G mutation at position 2063 is most common (*15*,*16*). Comparison of sequencing results with antimicrobial susceptibility testing confirmed that the mutation at positions 2063/2064 led to a high level of macrolide resistance (*15*). Thus, with the mutation detected, resistance of these strains can be assumed. We did not find macrolide resistance to be associated with a certain MLVA type, which confirms the findings of other reports (*16*), but the low number of resistant strains in our study is insufficient for us to draw a final conclusion.

The results of *Chlamydia* testing are remarkable for the comprehensive methodologic approach. In the past, the choice of diagnostic tests was usually limited: that is, either only *C. pneumoniae* was analyzed or all *Chlamydia* spp. identified were thought to be *C. pneumoniae*. Although the positivity of 1.4% for *C. pneumoniae* we found is in line with previous data from Germany (*22*), the proportions of positive samples of 2.1% for *C. psittaci* and 4.7% for all *Chlamydiae* spp. considered here deserve specific attention (Table 4). Chlamydial co-infection involving >2 *Chlamydia* spp. has been shown to be frequent in trachoma patients (*39*), but data from pneumonia patients remain scarce. Our finding that 19.3% of *Chlamydia*-positive patients harbored >2 *Chlamydia* spp. is in the same range as the 24% found in a recent trachoma study (*40*).

One unexpected finding in our study was that *C. psittaci* positivity could not be correlated with the patients having regular contact with birds at home or at work. Moreover, no seasonal patterns were identified. Nevertheless, our findings indicate that chlamydial species other than *C. pneumoniae* should be included in testing of CAP patients; in particular, *C. psittaci* should be included but also *C. trachomatis*. The relatively high prevalence of *C. psittaci* we found raises questions about its epidemiologic and etiologic importance, which should be addressed in future studies.

In summary, we confirmed a strong epidemic peak of *M. pneumoniae*–positive respiratory samples among adult patients with confirmed CAP in Germany during 2011–2012. During the fourth quarter of 2011, the incidence of infections temporarily rose to ≈27% of all patients investigated. *Chlamydia* spp. were found in 3.9% of samples, without epidemic peaks. Throughout the study period, the dominant *M. pneumoniae* P1 type was subtype 1; only 3.1% of *M. pneumoniae* strains were macrolide resistant. Both bacteria represent relevant pathogens in CAP, and awareness of their epidemiology, particularly among clinicians, is clearly warranted.
